# From the President’s desk: Part 3, 2026

**DOI:** 10.4102/safp.v68i1.6406

**Published:** 2026-08-27

**Authors:** Tasleem Ras

**Affiliations:** 1 Department of Family, Community and Emergency Care, Faculty of Health Sciences, University of Cape Town, Cape Town, South Africa; 2 President and Chair of Executive Committee, South African Academy of Family Physicians, Durbanville, South Africa

As we enter the second half of 2026, it is appropriate that we pause and reflect on what the first half of this year has brought us. The National Health Insurance (NHI) legislation is tied up in court, and the likelihood of a clear direction within the next 12 months seems dim. While we were still reeling from the shocking allegations about corruption within the Gauteng Health Department, the Madlanga Commission is laying bare some of the difficult and dark truths about the level that corruption has metastasised through our law enforcement apparatus. And, I am reliably informed that the country did not do well in the recent national cancer control survey, the results of which are yet to be published. For the Family Medicine community, this means that there is work to be done.

One of the ways that we can confront corruption on a grand scale is to team up with like-minded organisations, building alliances between civic and professional organisations that demand accountability and good governance. This also means that we can demand that our voices are heard when we start engaging on strategic matters that affect the development of our district, primary and community health platforms. To this end, our collaboration with rural partners in hosting the Rural and Family Physicians Joint Congress 2026 (https://saafp.org/about-us-2/conferences-2/2026_conference/), which features one of the most exciting range of speakers I have seen in a long time, is a step in the right direction as we build collaborative partnerships for primary care. Additionally, we recently had a very productive meeting with the South African Medical Association (SAMA) chief executive officer, Dr MT Nodikida, with the express intent of strengthening engagements around matters of strategic national importance.

While we are building alliances, the South African Academy of Family Physicians (SAAFP) has intentionally made its voice heard within the public domain on topical matters and will continue to do so. In the first half of this year, we published a statement on the ongoing man-made humanitarian disaster in Gaza as a result of the relentless attacks by the Israeli army on a civilian population. Additionally, we issued a strong statement on the xenophobic crisis that is still gripping our country. While some may say that these are political issues that do not affect the practice of family medicine, in reality the practice of medicine has always been deeply and significantly impacted by politics. In our ongoing struggle to realise health for all, we cannot avoid speaking truth to those in power.

One of the abiding challenges that newly graduated family physicians face is that of job security. The urgency to address this is being reinforced in the SAAFP president’s national registrar roadshow, which is currently underway. In the face of shrinking government expenditure on specialist positions, the situation is becoming even more dire. In a bid to be pro-active, the SAAFP leadership is actively engaging with private sector decision-makers to pave the way for those who may see private practice as a sustainable option. These engagements have been promising, but a considerable amount of work lies ahead if we are to be appropriately remunerated at specialist rates by private sector funders. Furthermore, heads of family medicine have met on 20 July 2026 to strategise on ways to engage with provincial and national departments of health when advocating for the funding of posts for registrars and consultants.

Our work does not occur in a South African vacuum, and for those of us who will be able to attend the 9th World Organisation of Family Doctors (WONCA) Africa conference in Gaborone, Botswana on 10–11 September 2026 (https://woncaafrica2026.org/), the achievements and challenges of African Family Medicine will be showcased. This links us with a global community of family physicians, along with a host of opportunities in academic, clinical and commercial ventures.

Although the road ahead seems challenging, I am always lifted by the spirit and competence of friends and colleagues within the family medicine community. It is in this sense of cohesion that we will find our strength and in the spirit of Ubuntu that we define and actualise health systems that work for all.

